# Maternal AGE Precursors During Lactation Alters Offspring Glycemic Homeostasis Early in Life

**DOI:** 10.3390/biology14020160

**Published:** 2025-02-05

**Authors:** Lucas P. J. Saavedra, Flávio A. Francisco, Scarlett R. Raposo, Keilah V. N. Cavalcante, Nilza C. Buttow, Stephanie C. Borges, Rodrigo M. Gomes, Hericles M. Campos, Gessica D. Gonçalves, Silvano Piovan, Paulo C. Ghedini, Kelly V. Prates, Ananda Malta, Paulo Matafome, Paulo C. F. Mathias, Douglas L. Almeida

**Affiliations:** 1 Department of Biotechnology, Genetics, and Cellular Biology, State University of Maringá, Maringá 87020-900, PR, Brazil; saavedralpj@gmail.com (L.P.J.S.); flvbio@hotmail.com (F.A.F.); scarlett_rr@hotmail.com (S.R.R.); ncbuttow@gmail.com (N.C.B.); stephaniecarvalhoborges@hotmail.com (S.C.B.); g.dutragoncalves@gmail.com (G.D.G.); silvanopiovan23@gmail.com (S.P.); kvp.86@hotmail.com (K.V.P.); nandamalt@hotmail.com (A.M.); pcfmathias@gmail.com (P.C.F.M.); 2 Department of Physiological Sciences, Federal University of Goiás, Goiânia 74690-900, GO, Brazil; keilah1506@gmail.com (K.V.N.C.); gomesrm@ufg.br (R.M.G.); 3 Department of Pharmacology, Federal University of Goiás, Goiânia 74690-900, GO, Brazil; hericlesmesquita@discente.ufg.br (H.M.C.); pcghedini@ufg.br (P.C.G.); 4 Institute of Physiology and Institute of Clinical and Biomedical Research, Faculty of Medicine and Center for Innovative Biomedicine and Biotechnology, University of Coimbra, 3000-447 Coimbra, Portugal; paulomatafome@gmail.com; 5 Coimbra Health School, ESTeSC, Instituto Politécnico de Coimbra, 3000-447 Coimbra, Portugal; 6 Clinical Academic Center of Coimbra, 3000-447 Coimbra, Portugal

**Keywords:** breastfeeding, developmental plasticity, advanced glycation end products, glycemia homeostasis, metabolism

## Abstract

Background: Advanced glycation end-products (AGEs) are linked to the development of oxidative stress, insulin resistance, and impaired insulin secretion. Adverse early life conditions, such as exposure to AGEs and their precursors, may lead offspring to the development of metabolic dysfunction in adulthood. Nonetheless, the early impact in offspring metabolism by maternal intake of AGEs precursors during lactation is not known. Objective: Investigate early life metabolism of the offspring whose breastfeeding dams were orally exposed to AGEs precursor. Methods: Breastfeeding Wistar rats were daily treated with the glycation precursor methylglyoxal (MG—60 mg/kg of bodyweight) by gavage or saline 0.9% control (CO) until weaning. In vivo glycemic homeostasis in male offspring was assessed, followed by euthanasia for tissue sample collection for ex vivo assessments. Results: At weaning, MG offspring presented decreased bodyweight (*p* < 0.05), perigonadal (*p* < 0.01) and retroperitoneal (*p* < 0.01) fat. MG offspring presented decreased glucose tolerance (*p* < 0.05), lower basal insulinemia (*p* < 0.001), reduced high-glucose static insulin secretion (*p* < 0.05), and reduced pancreatic islet area (*p* < 0.05). Accordingly, MG offspring pancreas showed lower GSH and SOD activity (*p* < 0.05; *p* < 0.001, respectively) and increased MPO (*p* < 0.05) activity. Conclusions: The consumption of AGE precursors by breastfeeding dams impaired offspring pancreatic function and glycemic homeostasis early in life.

## Simple Summary

Our research focused on how maternal exposure to dietary toxins during lactation influences the offspring’s physiological development. Breastfeeding Wistar rats were exposed to a compound called methylglyoxal (MG), a precursor to harmful substances known as advanced glycation end-products. Our data showed that the offspring from lactating MG faced difficulties in managing blood sugar levels, linked to lower insulin production, which is critical for maintaining proper glycemic control. Also, MG offspring pancreas, the only endogenous insulin source, showed signs of greater stress and reduced function in early life. These results reinforce the evidence that what a mother consumes while breastfeeding can significantly impact her child’s metabolic health, potentially increasing the risk of noncommunicable chronic diseases like diabetes in the future.

## 1. Introduction

Maintaining glucose homeostasis is a top priority for mammals, entailing a complex system of hormones, tissues, and organs committed to the task throughout life. Disrupted glucose homeostasis is associated with several pathologies, turning diabetes into the focal subject for much of the ongoing research in the biological and medical sciences. Meanwhile, diabetes and type 2 diabetes (T2D) still affect around 10% of the global population in the last decades [1,2]. Although the etiology for T2D and other metabolic disturbances is broad, including from genetics to lifestyle, solid data show the great influence of environmental stress on developmental phases of life for glucose homeostasis disruption [2,3].

Lactation is one of these periods for metabolic plasticity where environmental stresses drive long-term effects on health. Evidence shows that disturbances during the breastfeeding phase might influence a great deal for offspring adult phenotype, increasing the susceptibility for later metabolism-related diseases [4,5,6]. These metabolic disturbances include, but are not limited to, insulin resistance, impaired insulin secretion and progressive development of hyperglycemia, common features of T2D [6,7,8]. The onset of such conditions at an early age is a strong predictor for future metabolic illnesses.

Among several environmental stressors listed in the literature, solid data have highlighted the role of advanced glycation end-products (AGEs) in the physiopathology of T2D [9]. In short, elevated circulating levels of AGEs lead to β-cell apoptosis and insulin secretion deficiency [10,11,12]. Although high levels of AGEs and their precursors might be found in breastfeeding mothers and their infants, such as those observed in maternal diabetes or as an outcome of the high intake of ultra-processed foods, the early-in-life effects of such exposure on progeny glycemic homeostasis are not known [13]. Considering the role of environmental stresses at early life in the disruption of glycemic homeostasis and the metabolic disturbances induced by increased consumption of AGEs, in the present study we investigated the effects of breastfeeding dams’ intake of methylglyoxal (MG), an AGE precursor, on the glycemic homeostasis of male offspring during the breastfeeding period.

## 2. Materials and Methods

### 2.1. Experimental Design and Treatment

Male and female Wistar rats at reproductive age were kept under controlled temperature conditions (22 ± 2 °C) and photoperiod (12 h dark–light cycle). After 5 days of adaptation, animals were mated in a ratio of two females to one male. When pregnancy was detected, females were housed individually, and natural delivery was considered day 0 (PN0). At PN01, litters were standardized for eight pups per mother. Dams were divided into two groups: the control (CO), which received a saline solution (0.9% NaCl, 1 mL/kg of bodyweight [BW]); and methylglyoxal (MG), treated with methylglyoxal solution (60 mg/kg/day, Sigma-Aldrich^®^, São Paulo, SP, Brazil), the dose was selected because it has been shown previously that a dose ranging from 50 to 75 mg/kg/day is able to induce diabetic-like alterations in metabolism that resemble the diabetic animal model, Goto Kakizaki Rat [14,15,16,17]. Both treatments were administered daily by gavage during the lactation period (from PN1 to PN21). Animals were fed a standard chow (Nuvilab^®^ CR-1, Curitiba, Paraná, Brazil) and had free access to food and water throughout the experimental period. To follow the treatment evolution, the male offspring of each group were euthanatized and evaluated at 3 different standpoints (PN7, PN14, PN21) for biometric and biochemical assessment. Male offspring were chosen based on the gender differences in AGE metabolisms, with males being more susceptible to oxidative stress and inflammation induced by AGEs [18]. At least 4 different litters were used at each time point. All the animal handling, housing, and experimental procedures followed the guidelines of the Brazilian National Council for Control of Animal Experimentation (CONCEA) and were approved by the Ethic Commission in The Use of Animals (CEUA) from State University of Maringá, Brazil (protocol number 6345250918).

### 2.2. Euthanasia, Blood Collection, and Adiposity Evaluation

Dams from both groups had their bodyweight evaluated during lactation and were euthanized at weaning (PN21) for evaluation of periovarian, periuterin, retroperitoneal, and visceral fat depots. After 6 h of fasting, pups of each experimental timepoint (PN7, PN14, and PN21) were euthanized by decapitation using a rodent guillotine for blood sample collection. Blood samples were centrifuged (10,000 rpm for 5 min) for plasma collection and then frozen at −20 °C. Offspring retroperitoneal, perigonadal, and mesenteric visceral fat was removed and weighed at PN21.

### 2.3. Intraperitoneal Glucose Tolerance Test (ipGTT)

At the end of lactation (PN21) and after 6 hours of fasting, the litters were submitted for the intraperitoneal glucose tolerance test. They were weighed, and then the blood samples were collected by the tail to obtain fasting glycemia (time 0) by using a glycosometer (FreeStyle OptimumH^®^, Abbott Laboratories, São Paulo, Brazil). Afterwards, the pups received an intraperitoneal injection of glucose in the concentration of 2 g/kg of bodyweight. The blood glucose was evaluated at 15, 30, and 60 min after glucose administration. The AUC on the ipGTT was calculated.

### 2.4. Biochemical Analyzes

Basal glycaemia was quantified by the spectrophotometric glucose oxidase method (BIO200FL, Bio Plus^®^, São Paulo, SP, Brazil), using a commercial kit (Gold Analisa^®^, Belo Horizonte, MG, Brazil). Insulinemia was measured by radioimmunoassay (RIA) through a gamma particle emission counter (Wizard2AutomaticGammaCounter, TM-2470, PerkinElmer^®^, Shelton, CT, USA), using standard rat insulin, rat anti-insulin antibody (Sigma-Aldrich^®^, St. Louis, MO, USA), and 125 I-labeled recombinant human insulin (PerkinElmer^®^, Shelton, CT, USA) [19]. The values obtained in glycemia and insulinemia were used to calculate HOMA-β = [fasting plasma insulin (μIU/mL) × 360/(fasting plasma glucose (mg/dL) − 63], an indicator of beta-cell function, and also HOMA-IR = [fasting insulin (µIU/mL)] × [fasting glucose (mmol/L)]/22.5, an indicator of insulin resistance [20]. Total cholesterol was measured with the colorimetric cholesterol oxidase method, and the triglycerides were dosed by the colorimetric method of glycerol-3-phosphate oxidase using commercial kits (Gold Analisa^®^, Belo Horizonte/MG, Brazil). Both readings were carried out on spectrophotometry equipment (semi-automatic biochemical analyzer, BIO200FL, Bio Plus^®^, São Paulo, SP, Brazil). HDL-cholesterol was determined after chylomicrons and low-density lipoprotein precipitation with a commercial kit (Gold Analisa^®^, Belo Horizonte, MG, Brazil) and subsequent determination of HDL-cholesterol by the above-described method for total cholesterol dosage.

### 2.5. Pancreas Histology and Islet Morphology

Pancreas samples from PN7, PN14, and PN21 were fixed in 10% buffered formalin, dehydrated, embedded in histological paraffin, and sectioned (5 µm) in non-serial cuts. Tissue sections were deparaffinized, rehydrated, and stained with hematoxylin and eosin (H&E). Image capture was performed using a camera (QColor 3, Olympus^®^, Tokyo, Japan) attached to a light microscope. Analyses of the islet area were performed using 40 digital images (×400 magnification). Morphometric analyses were performed using Image-Pro Plus^®^ 4.5 software (Media Cybernetics, Silver Spring, MD, USA).

### 2.6. Pancreatic Islet Isolation and Static Insulin Secretion

Pancreatic islets were isolated by collagenase digestion (collagenase type V, 8 mg/mL, Sigma-Aldrich, St. Louis, MO, USA) and then selected with a microscope to exclude any contaminating tissues. For static incubation, groups of four islets from each group were incubated for 30 min in Krebs-Ringer Bicarbonate (KRB) buffer (115 mM NaCl, 5 mM KCl, 2.56 mM CaCl_2_, 1 mM MgCl_2_, 10 mM NaHCO_3_, 15 mM HEPES) supplemented with 16.7 mM glucose, 3 g of BSA/L, and equilibrated with a mixture of 95% O_2_/5% CO_2_, pH 7.4. This medium was then replaced with fresh KRB buffer, and the islets were incubated for a further 1 h with 16.7 mM glucose. Aliquots of the supernatant, taken at the end of the incubation period, were kept at −20 °C for posterior insulin measurement by RIA [21].

### 2.7. Oxidative Stress and Inflammation

The activity of SOD was evaluated according to the protocol of Marklund and Marklund (Marklund & Marklund, 1974), based on its ability to inhibit the auto-oxidation of pyrogallol. Tris-HCL buffer (1 mM) and ethylenediaminetetraacetic acid (EDTA; 5 mM, pH 6.5) were added to each sample, and the reaction was started with 1 mM pyrogallol and incubated at 25 °C for 20 min. The reaction was stopped with 1 M hydrochloric acid. The samples were centrifuged at 14,000× *g* for 4 min, and the absorbance of each supernatant was read at 405 nm. The amount of protein that inhibited the reaction by 50% was equal to 1 unit (U) of SOD activity. The results are expressed as units (U) of SOD/mg of protein [22].

The activity of CAT was analyzed by adding a supernatant solution that contained 30% H_2_O_2_ and 0.1 M Tris-HCL buffer (pH 8.5) and distilled water according to Aebi (Aebi, 1984). Readings were performed at a wavelength of 240 nm over 5 min. The results are expressed as mmol/min/mg of protein [23]. The reduced glutathione (GSH) analysis was performed by homogenizing the tissue in 200 mM potassium phosphate buffer (pH 6.5). The reaction of GSH with 5,5′-dithiobis-2-nitrobenzoic acid was read at 412 nm. Individual values were interpolated based on a GSH standard curve and are expressed as μg of GSH/g of tissue. For lipid hydroperoxide (LOOH) evaluation, the other part of the homogenate was centrifuged for 20 min at 9000× *g*, and part of the supernatant was used to determine lipid hydroperoxide (LOOH) levels. Readings were performed at 560 nm using a spectrophotometer. LOOH concentrations were determined using an extinction coefficient of 4.3 mmolar 1/cm, and the results are expressed as mmol/mg of tissue. For myeloperoxidase (MPO) enzyme activity, the precipitate from tissue homogenate centrifugation was resuspended in 80 mM potassium phosphate buffer that contained 0.5% hexadecyltrimethylammonium. The samples were homogenized and centrifuged for 20 min at 11,000× *g* at 4 °C. The reaction was performed in a 96-well plate using tetramethylbenzidine. Enzymatic activity of myeloperoxidase (MPO) was determined at 620 nm using a spectrophotometer. The results are expressed as units of optical density (OD)/min/mg of protein.

### 2.8. Statistical Analyzes

The results were presented as mean ± standard error of the mean (SEM). The data were analyzed through Student’s *t*-test for parametric data and Mann–Whitney for non-parametric. Values of *p* < 0.05 were considered significant. The tests were conducted using GraphPad Prisma Software version 7.00 for Windows (GraphPad Software Inc., La Jolla, CA, USA).

## 3. Results

### 3.1. MG Intake on Dams Murinometric Profile

No significant differences were observed between CO and MG dams bodyweight gain, food intake, or visceral adiposity (Table 1) during lactation, indicating that MG oral intake was not able to induce murinometric changes in the breastfeeding dams.

### 3.2. Effects of Maternal MG Intake in Offspring Bodyweight, Adiposity, and Glycemic Homeostasis

**Figure 1:**
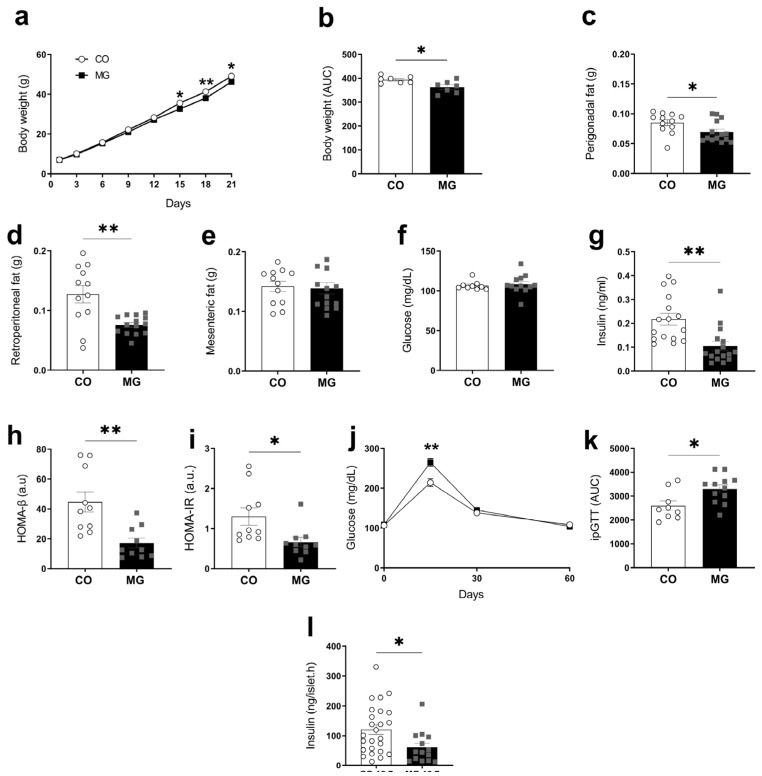
Maternal exposure to MG leads to decreased fat mass and decreases insulin secretion. Biometric parameters, visceral adiposity and biochemical parameters of male offspring during lactation. Bodyweight gain evolution (**a**); AUC of bodyweight evolution (**b**); Perigonadal fat (**c**); Retroperitoneal fat (**d**); Mesenteric fat (**e**); Basal plasma glucose (**f**); Basal plasma insulin (**g**); β-cell function, HOMA-β (**h**); Insulin resistance, HOMA-IR (**i**); Glycemia during intraperitoneal glucose tolerance test (**j**); AUC of glucose tolerance test (**k**); Static insulin secretion on isolated pancreatic islets in a post-prandial glucose concentration (**l**); (CO, n = 5–6; MG, n = 5–6). Data are presented as mean ± SEM. To compare the experimental groups Student’s *t*-test was used, where * *p* < 0.05 and ** *p* < 0.01.

**Figure A1:**
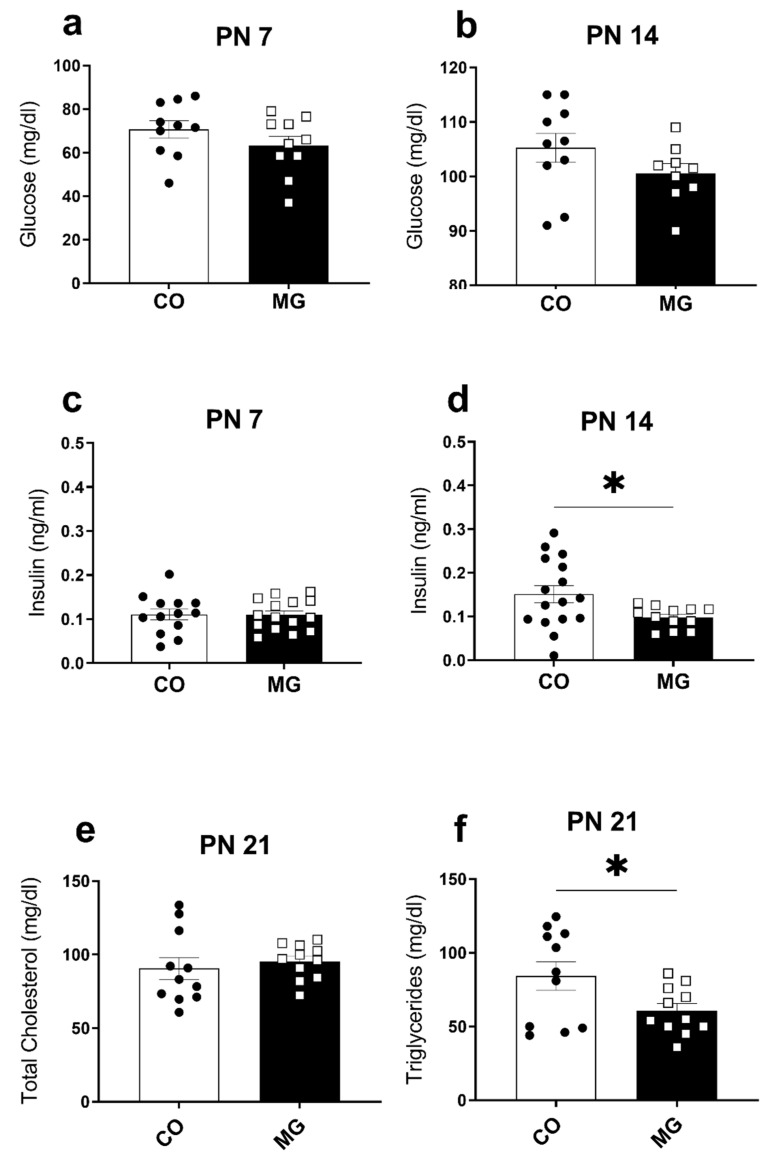
Offspring biochemical plasmatic parameters. Plasmatic glucose at post-natal (PN) day 7 (**a**); Plasmatic glucose at PN14 (**b**); Basal insulin at PN7 (**c**); Basal insulin at PN14 (**d**); Total cholesterol at PN21 (**e**); Plasmatic triglycerides (**f**). (CO, n = 12–16; MG, n = 14–16). Data are presented as mean ± SEM. To compare the experimental groups Student’s *t*-test was used, where * *p* < 0.05.

Differently from that observed for dams, maternal MG consumption affected neonatal rats’ development when compared to CO animals. At PN21, MG offspring showed reduced bodyweight gain (CO 393.9 ± 5.5092 vs. MG 327.5 ± 9.520, *p* < 0.05, Figure 1b), decreased perigonadal (CO 0.0850 ± 0.0049 vs. MG 0.0695 ± 0.0047, *p* < 0.01, Figure 1c) and retroperitoneal fat weight (CO 0.1271 ± 0.0114 vs. MG 0.07599 ± 0.0040, *p* < 0.01, Figure 1d), evidencing lower adiposity. There was a reduction of basal insulinemia in MG offspring at PN14 (CO 0.1513 ± 0.0795 vs. MG 0.0983 ± 0.0248, *p* < 0.05, Figure A1d) that was sustained until PN21 (CO 0.2174 ± 0.0244 vs. MG 0.1046 ± 0.0195, *p* < 0.01, Figure 1g). Also, it was observed that β-cell function decreased (CO 44.66 ± 6.71 vs. MG 17.18 ± 3.30, *p* < 0.01, Figure 1h), HOMA-IR decreased (CO 1.30 ± 0.22 vs. MG 0.66 ± 0.12, *p* < 0.05, Figure 1i), as well as the glucose intolerance at time 15 of GTT (CO 216.4 ± 12.39 vs. MG 264.5 ± 9.64, *p* < 0.01, Figure 1j) and the area under the curve (CO 2594 ± 204.1 vs. MG 3299 ± 167.7, *p* < 0.01, Figure 1k). MG increased static insulin secretion on isolated pancreatic islets in a post-prandial glucose condition (CO 120.7 ± 16.27 vs. MG 60.51 ± 14.30, *p* < 0.05, Figure 1l). Collectively, our data show that maternal exposure to MG leads to impairment of glycemic homeostasis and hypoinsulinemia.

### 3.3. Effects of Maternal Consumption of MG on Offspring Pancreatic Islet Morphology

**Figure 2:**
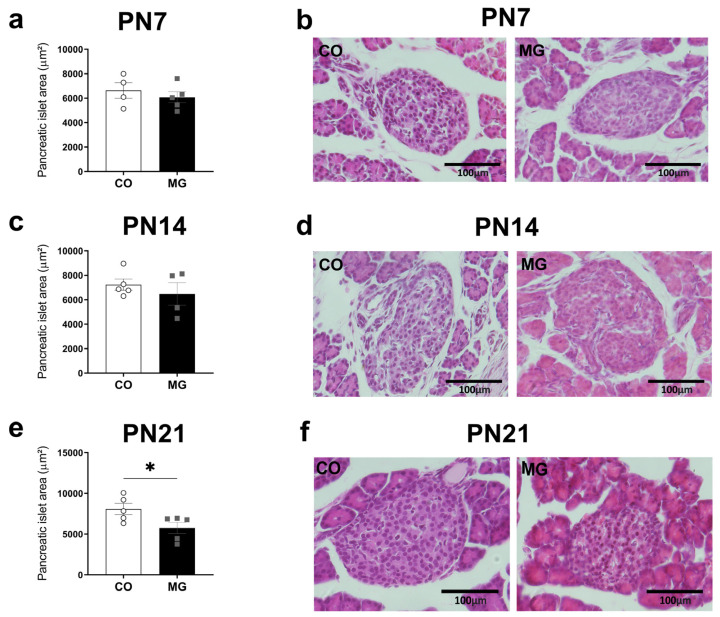
Maternal exposure to MG alters pancreatic islet morphology. Pancreatic islet area at post-natal day (PN7) (**a**); Representative images of CO and MG islets at PN7 (**b**); Pancreatic islet area at PN14 (**c**); Representative images of CO and MG islets at PN14 (**d**); Pancreatic islet area at PN21 (**e**); Representative images of CO and MG islets at PN14 (**f**); (CO, n = 4–5; MG, n = 5). Data are presented as mean ± SEM. To compare the experimental groups, Student’s *t*-test was used, where * *p* < 0.05.

The pancreatic islet area at PN21 was reduced in MG offspring (CO 8086 ± 690.1 vs. MG 5741 ± 674.8, *p* < 0.05, Figure 2e); no difference was observed at PN7 or PN14 (Figure 2a,c). Maternal MG exposure prejudices with proper pancreatic islet development in male offspring.

### 3.4. Effects of Maternal Consumption of MG on Offspring Pancreatic Oxidative Stress and Inflammation

**Figure 3:**
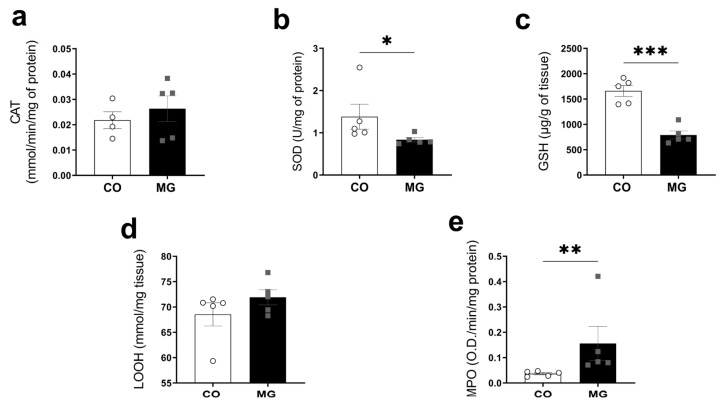
Maternal exposure to MG leads to offspring pancreatic oxidative stress and inflammation. Catalase—CAT activity (**a**); Superoxide dismutase—SOD activity (**b**); Reduced glutathione—GSH (**c**); Lipid hydroperoxide—LOOH (**d**); Myeloperoxidase—MPO (**e**); (CO, n = 4–5; MG, n = 5). Data are presented as mean ± SEM. To compare the experimental groups Student’s *t*-test was used, where * *p* < 0.05, ** *p* < 0.01, and *** *p* < 0.001.

Superoxide dismutase activity (SOD; CO 1.381 ± 0.296 vs. MG 0.835 ± 0.114, *p* < 0.05, Figure 2b) and glutathione activity (GSH, CO 1661 ± 107.4 vs. MG 791.3 ± 80.43, *p* < 0.001, Figure 2c) were reduced in MG offspring, while myeloperoxidase, a biomarker for inflammation (MPO, CO 0.037 ± 0.004 vs. MG 0.156 ± 0.066, *p* < 0.01, Figure 2e), was increased in these animals, compared to the CO group. No difference was observed in CAT or LOOH (Figure 3a,d). Collectively, our data show that MG exposure during lactation leads to reduced islet area and increased pancreatic oxidative stress.

### 3.5. Effects of Maternal Consumption of MG on Offspring Liver Oxidative Stress and Inflammation

**Figure 4:**
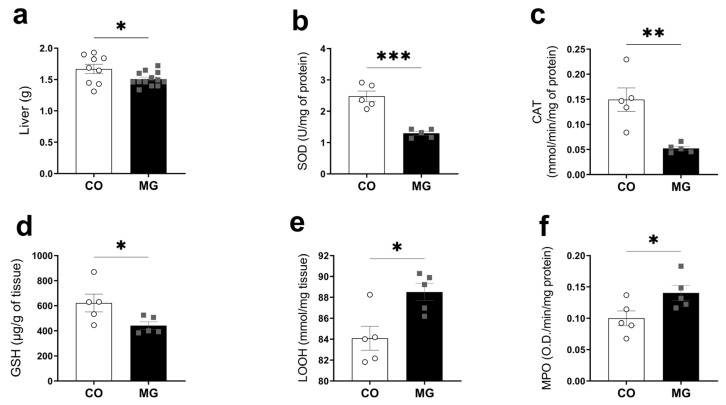
Maternal exposure to MG leads to offspring liver oxidative stress and inflammation. Liver mass (**a**); Superoxide dismutase—SOD activity (**b**); Catalase—CAT activity (**c**); Reduced glutathione—GSH (**d**); Lipid hydroperoxide—LOOH (**e**); Myeloperoxidase—MPO (**f**); (CO, n = 4–5; MG, n = 5). Data are presented as mean ± SEM. To compare the experimental groups Student’s *t*-test was used, where * *p* < 0.05, ** *p* < 0.01, and *** *p* < 0.001.

Maternal MG exposure during breastfeeding reduced liver mass (CO 1.67 ± 0.08 vs. MG 1.51 ± 0.031, *p* < 0.05, Figure 4a), superoxide dismutase activity (SOD; CO 2.48 ± 0.17 vs. MG 1.3 ± 0.06, *p* < 0.001, Figure 4b), catalase activity (CAT; CO 0.15 ± 0.02 vs. MG 0.05 ± 0.01, *p* < 0.01, Figure 4c), and reduced glutathione (GSH, CO 621.9 ± 70.73 vs. MG 442.1 ± 30.49, *p* < 0.05, Figure 4d), while lipid peroxidation (LOOH, CO 84.09 ± 1.15 vs. MG 88.52 ± 0.81, *p* < 0.05, Figure 4e) and myeloperoxidase, a biomarker for inflammation (MPO, CO 0.10 ± 0.01 vs. MG 0.14 ± 0.02, *p* < 0.05, Figure 2f), were increased. Collectively, our data show that MG exposure during lactation leads to increased liver oxidative stress and inflammation.

## 4. Discussion

The consumption of AGEs and their precursors has been linked to impaired metabolic health in humans and in animal models [14,17,24,25]. Here, for the first time, we show that breastfeeding dam’s consumption of MG, AGEs precursor, leads the offspring to the onset of early life glucose intolerance and impaired basal and stimulated insulin secretion. Even though the dams themselves were not affected by the MG consumption in their biometrics (Table 1). Interestingly, the changes in glucose metabolism were accompanied by impaired pancreatic β-cell development, as the data reveals that maternal consumption of MG decreased offspring pancreatic islet area and reduced the pancreatic antioxidant defenses, with an increment of inflammatory biomarkers.

In rats, adipogenesis occurs primarily in the last week of gestation and persists through lactation until weaning [4]. MG chronic exposure impairs adipose tissue expansion by reducing angiogenesis in the tissue, which in turn leads to hypoxia, inflammation, impaired insulin signaling, and augmented release of fatty acids [14,15]. These data found in the literature help us to understand the reduced visceral fat deposition and lower TG levels in MG male offspring at PN21 in this study. Moreover, hormones such as insulin, which we observed to be reduced, along with transcription factors, like C/EBP and PPARγ, exert trophic effects in adipose tissue, being important to the normal tissue development [26,27,28].

Besides the decreased visceral fat, MG offspring, exposed to glycation exclusively during (PN1 to PN21) and via breastfeeding, also showed decreased bodyweight. Animal studies have shown that early-life growth impairment may be followed by fat tissue expansion and accumulation later in life [29]. At PN21, MG animals, exposed to glycation exclusively during breastfeeding (PN1 to PN21), showed decreased bodyweight and visceral fat. Similarly, humans born small for their gestational age also pass through the process of catch-up growth, where they accumulate more fat [30]. Therefore, it is reasonable to suggest that the reduced growth observed here likely leads to a later catch-up growth.

The growth restriction in the offspring from breastfeeding dams exposed to MG at PN21 is not limited to the bodyweight and bodyfat but is also seen in the pancreatic islet. The neonatal period is also a phase of intense pancreatic development, with increased growth of insulin-positive cells, namely β cells, accompanied by increased circulating insulin levels [31,32,33,34]. In the present study, we identified in the offspring glucose intolerance, low levels of plasma insulin, decreased static insulin secretion in high levels of glucose, and decreased pancreatic islet areas, confirming the hypothesis that neonatal exposure to glycotoxins may impair endocrine pancreas development. It is important to highlight that MG is a precursor for AGE formation, and its administration promotes increased circulating AGEs [12,17,35,36,37], and β cells exposed to AGEs, in vitro and in vivo, exhibit increased apoptosis and impaired insulin secretion [37,38,39].

Chronic exposition to MG decreases the expression of activity of key antioxidant enzymes, such as CAT, SOD, and GPX, also being observed with reduced GSH levels and increased generation of reactive oxygen species, which activate MAPKs and increase UCP-2 levels, leading to β-cell damage and dysfunction [38,39]. In the present study we show reduced SOD and CAT activity confirming previous results obtained with adult rats [40,41], suggesting a possible mechanism involved in the long-term β cell pathophysiology. However, we have to highlight that despite decreased activity of the antioxidant enzymes, we did not observe any increase in the oxidative stress marker, LOOH (Figure 3d); however, we observed an increase in inflammatory marker MPO (Figure 3e). We have found that MG offspring had decreased GSH levels. GSH is an important factor for MG detoxification since the glyoxalase system uses GSH to convert MG to lactate [42]. Previous studies have reported that MG decreased GSH in the liver [41]. Similarly, we have observed increased oxidative stress and GSH reduction in the liver of the offspring (Figure 4d). Maternal glycation-induced inhibition of the enzyme that decomposes MG, glyoxalase I, increases the levels of MG-H1 in the offspring, an AGE derived from a reaction between MG and the amino acid arginine [43]. Also, it was found that there was decreased total antioxidant capacity in breastmilk, showing evidence that maternal glycation may alter offspring metabolism through alterations in breastmilk composition [43]. In fact, several studies have found that maternal adverse health conditions during lactation, such as obesity or diabetes, is related to alterations in breastmilk composition and, lastly, with negative offspring outcomes such as higher adiposity, poor glycemic control, and low-grade inflammation [5]. 

MG or AGEs exposure occurs through endogenous or external sources. AGEs are formed in foods that undergo high cooking temperatures, such as frying, grilling, and roasting; ultra-processed foods are particularly rich in these compounds [44,45]. Infant formula, as an ultra-processed product has high levels of AGEs, and t intake during infancy may increase infant exposure to these compounds [46,47,48,49]. High glucose levels during diabetes increase endogenous formation of MG and AGEs, being implicated in the pathophysiology of diabetes [9,50,51]. Smoking has been shown to be related to increased AGEs levels [52,53]. Maternal smoking was shown to increase skin AGEs accumulation, which was positively associated with babies’ skin AGEs [54]. Thus, some interventions that may be taken to prevent MG and AGEs exposure during perinatal life include proper glycemic control in mothers with diabetes, the adoption of a balanced diet low in ultra-processed food, quitting smoking, and exclusively breastfeeding during the first 6 months [13].

Although maternal transfer of AGE precursors is the likely mediator of offspring phenotype, we cannot rule out that the effects are indirectly mediated through other maternal metabolic signals, since we were unable to measure MG levels in plasma or milk of dams. Another caveat is the fact that we only evaluated male offspring, since there are gender differences in the metabolism, with males being more susceptible to oxidative stress and inflammation induced by AGEs [18].

Although the present study lacks data measuring the content of MG and AGEs in plasma and milk, the novel findings show the negative effects of oral intake of MG and glycation by breastfeeding Wistar rats on offspring glycemic homeostasis early in life, likely an outcome of the impaired pancreas development. As is often reported and already discussed, such early-life metabolism impairment is likely to persist in adult life, predisposing to a diabetic phenotype. In this sense, the present results corroborate the concept of developmental origins of health and disease (DOHaD), reinforcing that during critical phases of development (e.g., pre-conception, gestation, lactation, and adolescence), the mammals are vulnerable to environmental changes that may increase the risk of developing cardiometabolic diseases in adulthood [7].

## 5. Conclusions

The consumption of MG by breastfeeding dams impaired offspring glucose homeostasis from early developmental age, mainly by affecting the endocrine pancreas development. Pancreatic islets were smaller, showed poor oxidative capacity, and had impaired insulin secretion. The low circulating levels of insulin might have reduced its full trophic effect, leading MG male offspring to lower visceral adipose tissue accompanied by reduced bodyweight gain for the age, signs of impaired early growth. Importantly, these early alterations may lead to a diabetic phenotype in adulthood [22]. This study highlights the urge for clinical and epidemiological research investigating whether maternal consumption of AGEs and their precursors during the perinatal period, or increased AGEs circulating levels as in maternal diabetes, may predispose their progenies to alterations in glycemic homeostasis early in life.

## Figures and Tables

**Table 1:** Biometric parameters of dams during lactation and visceral fat at weaning. (CO, n = 5–6; MG, n = 5–6). Data are presented as mean ± SEM. To compare the experimental groups Student’s *t*-test was used.

Parameter	CO	MG	*p*
AUC Bodyweight evolution (A.U.)	271.5 ± 55.32	192.5 ± 30.65	n.s.
AUC Food intake (A.U.)	478.8 ± 44.87	456.9 ± 4.49	n.s.
Retroperitoneal fat (g)	2.19 ± 0.24	2.04 ± 0.38	n.s.
Periovarian fat (g)	1.16 ± 0.16	1.01 ± 0.17	n.s.
Periuterin fat (g)	2.85 ± 0.24	2.50 ± 0.74	n.s.

## Data Availability

Data described in the manuscript, code book, and analytic code will be made available upon request pending application and approval.
